# P03-018 - Diversity in presenting manifestations of AUTOINFL

**DOI:** 10.1186/1546-0096-11-S1-A216

**Published:** 2013-11-08

**Authors:** S Boiu, B Neven, S Compeyrot-Lacassagne, R Mouy, C Wouters, B Bader-Meunier, M Gattorno, P Quartier

**Affiliations:** 1 Pediatric Immunology, Hematology and Rheumatology Unit, Necker-Enfants Malades Hospital, Assistance Publique Hôpitaux de Paris, Paris, France; 2 Paris-Descartes University and IMAGINE Institute, Paris, France; 3 Departement of Pediatric Rheumatology, Gaslini Institute, Genoa, Italy

## Introduction

The autoinflammatory diseases (AID) include monogenic and polygenic disorders characterized by primary dysfunction of the innate immune system.

## Objectives

To describe the clinical spectrum, genetic background and therapy in a cohort of AID patients followed in a reference Pediatric Rheumatology center.

## Methods

Medical records of AID patients followed between May 2007 and November 2010 and entered in the Eurofever Registry were studied.

## Results

Fifty six patients were included: 17 Cryopyrin-Associated Periodic Syndromes (CAPS), 4 TNF-Receptor-Associated Periodic fever Syndrome (TRAPS), 5 Hyperimmunoglobulinaemia D with periodic fever Syndrome (HIDS), 18 Familial Mediterranean Fever (FMF), 6 Chronic Recurrent Multifocal Osteomyelitis (CRMO), 2 Synovitis, Acne, Pustulosis, Hyperostosis, Osteitis (SAPHO) syndrome and 4 Behçet’s Disease (BD). The median follow-up was 2 years (0-14). The male/female ratio was 20/36. The median age was 2.5 years at disease onset and 4 at diagnosis. Family history was positive in 34% of patients. Clinical manifestations included fever (79%), musculoskeletal (77%), gastrointestinal (63%), mucocutaneous (61%), neurological (41%), ocular (34%), cardiorespiratory (13%), and genitourinary (2%) findings, lymphadenopathy with/or hepatosplenomegaly (16%) and growth impairment (25%). Complications/sequelae developed in 45% of patients. Six patients presented with unusual manifestations: neonatal peritonitis (1 CAPS), pancreatitis (1 TRAPS), acute glomerulonephritis (1 FMF), complicated Henoch -Schönlein purpura (1 FMF), peritoneal adhesions with intestinal occlusion (1 FMF), periorbital pain (1 CRMO) and cerebral thrombosis (1 BD). AID was associated with other diseases in 2 patients (FMF/Henoch-Schönlein purpura and CRMO/enthesitis-related arthritis). One mutant allele was found in 16/17 CAPS, 4/4 TRAPS and 4/18 FMF patients. Two mutant alleles were present in 5/5 HIDS and 11/18 FMF patients. The most used therapeutic agents were biologics (54%) (Anakinra, Canakinumab, Etanercept, Adalimumab), NSAIDs (48%), colchicine (45%) and corticosteroids (29%). Anti-interleukin-1 therapy and colchicine proved efficacy in CAPS and FMF patients, respectively. In addition, favorable responses demonstrated anti-interleukin-1 therapy in TRAPS, HIDS and colchicine-resistant FMF patients, as well as Etanercept in TRAPS, HIDS and CRMO patients non-responsive to NSAIDs. 57% and 41% of patients were in complete and partial remission, respectively, at last visit.

## Conclusion

AID in children are associated with a broad spectrum of manifestations. Early diagnosis and referral are essential as efficient therapy can be proposed in most cases.

## Competing interests

None Declared.

